# Long-Term Renal Function after Catheter Ablation of Atrial Fibrillation

**DOI:** 10.3390/jcdd10040151

**Published:** 2023-03-31

**Authors:** Vladan Kovačević, Milan M. Marinković, Aleksandar Kocijančić, Nikola Isailović, Jelena Simić, Miroslav Mihajlović, Vera Vučićević, Tatjana S. Potpara, Nebojša M. Mujović

**Affiliations:** 1 Cardiology Clinic, University Clinical Center of Serbia, 11000 Belgrade, Serbia; 2 Faculty of Medicine, University of Belgrade, 11000 Belgrade, Serbia; 3 Center for Anesthesiology, University Clinical Center of Serbia, 11000 Belgrade, Serbia

**Keywords:** atrial fibrillation, catheter ablation, late recurrence of atrial arrhythmia, renal function, chronic kidney disease, estimated glomerular filtration rate

## Abstract

**Background:** Atrial fibrillation (AF) is associated with the development and progression of chronic kidney disease (CKD). This study evaluated the impact of long-term rhythm outcome after catheter ablation (CA) of AF on renal function. **Methods and results:** The study group included 169 consecutive patients (the mean age was 59.6 ± 10.1 years, 61.5% were males) who underwent their first CA of AF. Renal function was assessed by eGFR (using the CKD-EPI and MDRD formulas), and by creatinine clearance (using the Cockcroft–Gault formula) in each patient before and 5 years after index CA procedure. During the 5-year follow-up after CA, the late recurrence of atrial arrhythmia (LRAA) was documented in 62 patients (36.7%). The mean eGFR, regardless of which formula was used, significantly decreased at 5 years following CA in patients with LRAA (all *p* < 0.05). In the arrhythmia-free patients, the mean eGFR at 5 years post-CA remained stable (for the CKD-EPI formula: 78.7 ± 17.3 vs. 79.4 ± 17.4, *p* = 0.555) or even significantly improved (for the MDRD formula: 74.1 ± 17.0 vs. 77.4 ± 19.6, *p* = 0.029) compared with the baseline. In the multivariable analysis, the independent risk factors for rapid CKD progression (decline in eGFR > 5 mL/min/1.73 m^2^ per year) were the post-ablation LRAA occurrence (hazard ratio 3.36 [95% CI: 1.25–9.06], *p* = 0.016), female sex (3.05 [1.13–8.20], *p* = 0.027), vitamin K antagonists (3.32 [1.28–8.58], *p* = 0.013), or mineralocorticoid receptor antagonists’ use (3.28 [1.13–9.54], *p* = 0.029) after CA. **Conclusions:** LRAA after CA is associated with a significant decrease in eGFR, and it is an independent risk factor for rapid CKD progression. Conversely, eGFR in arrhythmia-free patients after CA remained stable or even improved significantly.

## 1. Introduction

Atrial fibrillation (AF) is the most common sustained cardiac arrhythmia in adults, with a prevalence between 2% and 4%. Patients with AF have an increased risk of mortality, stroke, and heart failure [1].

Chronic kidney disease (CKD) is defined as an estimated glomerular filtration rate (eGFR) < 60 mL/min/1.73 m^2^ present for >3 months [2]. The prevalence of CKD and AF increases with age. These two diseases share several risk factors such as obesity, hypertension, diabetes mellitus, coronary artery disease, and heart failure [3,4]. Previous observational studies showed that AF and CKD have a bidirectional relationship. The presence of AF is associated with the development and progression of CKD, while the presence of CKD increases the risk of new-onset AF [5,6,7,8].

Catheter ablation (CA) of AF is more effective than antiarrhythmic drugs (AADs) for long-term rhythm control [9]. Long-term success of AF ablation including multiple procedures varies from 50 to 80% [10].

The aim of this study was to evaluate changes in the parameters of renal function after AF ablation during a 5-year follow-up and the impact of the long-term post-ablation rhythm outcome on renal function.

## 2. Materials and Methods

### 2.1. Study Population

This prospective, single-center study included consecutive adult patients (*n* = 187) who underwent their first CA for symptomatic AF refractory to at least one Class Ic or III AAD between January 2016 and December 2017 at the Cardiology Clinic, University Clinical Centre of Serbia. The major exclusion criteria were a previous AF ablation, age > 80 years, and end-stage renal disease (ESRD). Patients whose serum creatinine (sCr) values were not available at the end of the study (*n* = 18) were excluded from further analysis. Therefore, the final study group included 169 patients.

### 2.2. Pre-Ablation Work Up

All patients received oral anticoagulant therapy at least 6 weeks before CA. Class Ic AADs were discontinued 3–5 days before the admission, while amiodarone was stopped more than 1 month prior to CA.

Paroxysmal AF (PAF) was defined as an AF episode lasting longer than 30 s but shorter than 7 days. Nonparoxysmal AF (NPAF) included both persistent and long-standing persistent AF lasting >7 days to 1 year or >1 year, respectively [9].

Data on AF history, comorbidities, and previous drug therapy were collected from medical records. Before the procedure, all patients underwent transthoracic echocardiography, 24 h Holter monitoring, and chest-computed tomography.

### 2.3. Evaluation of Renal Function

Renal function was evaluated in all patients on hospital admission, before CA. We used the Chronic Kidney Disease Epidemiology Collaboration (CKD-EPI) formula to estimate GFR. According to Kidney Disease: Improving Global Outcome (KDIGO) recommendations, we divided patients into four groups: (1) normal kidney function, eGFR ≥ 90 mL/min/1.73 m^2^; (2) mildly decreased kidney function, eGFR 60–89 mL/min/1.73 m^2^; (3) mild to moderate CKD, eGFR 30–59 mL/min/1.73 m^2^; and (4) severe CKD, eGFR 15–29 mL/min/1.73 m^2^. Patients with eGFR < 15 mL/min/1.73 m^2^ were excluded from the study.

For each patient, in addition to the CKD-EPI formula, we also calculated eGFR using the Modification of Diet in Renal Disease (MDRD) formula and creatinine clearance (CrCl) using the Cockcroft–Gault (CG) formula.

### 2.4. Ablation Procedure

The AF ablation strategy in our hospital has already been described in detail elsewhere [11]. Briefly, after the transseptal puncture, the anatomical map of the left atrium (LA) was created (Ensite Velocity/Precision; St. Jude Medical, St. Paul, MN, USA) and fused with its computed tomography model. Cardiac CT scan was performed the day before the procedure using 100 mL of non-ionic contrast iohexol. Radiofrequency (RF) point-by-point ablation was performed with the 4mm externally irrigated tip ablation catheter (Cool Flex; St. Jude Medical, St. Paul, MN, USA) navigated by a deflectable long sheath (Agilis NT; St. Jude Medical, St. Paul, MN, USA) with a power limit of 25 to 30 W and flow rate of 17 mL/min. Local RF delivery was continued until a >80% reduction of atrial potential amplitude or for a maximum of 20 to 30 s and 40 to 60 s at the posterior and anterior LA wall, respectively. Ipsilateral pulmonary veins (PVs, left and right) were encircled in pairs with a circumferential ablation line deployed 5 mm from the anterior and 15 to 20 mm from the posterior aspects of their ostia, respectively. During the ablation, the residual electrical activity of PVs was evaluated with a duodecapolar circumferential mapping catheter.

Patients with PAF underwent a circumferential antral pulmonary vein isolation (PVI), as a stand-alone ablation strategy. In patients with NPAF, on top of PVI, additional LA substrate modification was performed. Linear LA ablation (the roof line plus mitral isthmus ablation) and/or complex fragmented LA electrograms ablation was performed in 33 (19.5%) and 14 (8.3%) patients, respectively. Ablation of typical atrial flutter (AFL) was performed in 44 (26%) patients. At the index procedure, femoral artery pseudoaneurysm and cardiac tamponade occurred in one and three patients, respectively. There were no major complications related to repeated CA procedures.

### 2.5. Post-Ablation Follow-Up

The AAD and oral anticoagulant (OAC) used before the procedure were administered during the 3-month blanking period post-CA in all patients. Afterwards, the oral anticoagulant was continued only in those with a CHA_2_DS_2_VASc score of >1, while AADs were discontinued in all patients.

Rhythm monitoring during follow-up consisted of 24 h Holter recordings at discharge; 1, 3, and 6 months post-CA; and every 6 months thereafter. Atrial arrhythmia recurrence was defined as any (symptomatic or asymptomatic) documented atrial arrhythmia post-CA, such as AF, atrial tachycardia (AT), or AFL, lasting more than 30 s. A recurrence recorded within the 3-month blanking period after CA was defined as early recurrence of atrial arrhythmia (ERAA), while recurrence after the 3-month blanking period was classified as late recurrence of atrial arrhythmia (LRAA). Patients experiencing LRAA were offered AAD therapy or repeat CA.

Renal function (eGFR using CKD-EPI and MDRD, and CrCl using the CG formula) was evaluated for each patient 5 years after the initial CA procedure. Based on KDIGO recommendations, a change in GFR category combined with a 25% or greater change in eGFR from the baseline measurement was defined as a certain rise or drop. Patients who experienced a change in GFR category combined with a less than 25% change in eGFR were categorized as having an uncertain rise or drop. Patients who did not experience a change in GFR category were considered as stable. Rapid CKD progression was defined as a decline in eGFR > 5 mL/min/1.73 m^2^/year.

### 2.6. Statistical Analysis

Continuous variables with normal distribution were presented as mean and standard deviation (SD), while those with asymmetrical distribution were reported as median and interquartile range (IQR). Categorical variables were shown as numbers and percentages. Continuous variables were compared using an independent samples *t*-test, whereas the difference between categorical variables was analyzed using χ^2^ or Fisher’s exact test. Paired samples *t*-test was used to compare continuous variables before and after CA, while proportions were compared using the McNemar test. Linear regression was used to analyze the association between 5-year eGFR change (ΔeGFR) and the rhythm outcome after CA. Risk factors for rapid progression of CKD were evaluated using univariate and multivariate Cox regression analysis. A two-sided *p*-value of <0.05 was considered statistically significant. All analyses were conducted using SPSS software (version 26.0, IBM, Armonk, New York, NY, USA).

## 3. Results

### 3.1. Study Population

**Figure 1:**
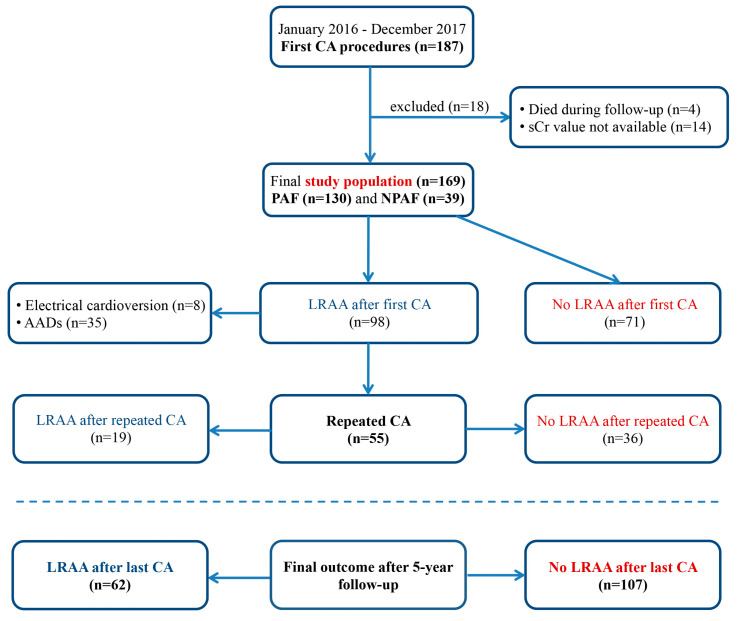
Study flow-chart and rhythm outcome after 5-year follow-up. AADs, antiarrhythmic drugs; CA, catheter ablation; LRAA, late recurrence of atrial arrhythmia; PAF, paroxysmal atrial fibrillation; NPAF, nonparoxysmal atrial fibrillation; sCr, serum creatinine.

The study group included 169 patients (the mean age was 59.6 ± 10.1 years, 61.5% were males). During the 5-year follow-up, LRAA was detected in 98 patients (58%) after the index procedure. Repeated ablation was performed in 55 patients (32.5%). Finally, LRAA was documented in 62 patients (36.7%) after their last CA, while 107 patients (63.3%) maintained sinus rhythm (Figure 1).

The baseline clinical characteristics of the study population are presented in detail in Table 1. Prior to CA, PAF and NPAF were documented in 130 (76.9%) and 39 (23.1%) patients, respectively. The most common comorbidities included hypertension (69.2%), diabetes (10.1%), and coronary artery disease (7.7%). Patients with LRAA after the last CA had a significantly higher prevalence of NPAF, larger LA, and higher CHA_2_DS_2_-VASc score at baseline compared with those who maintained sinus rhythm. There was no significant difference in other clinical characteristics prior to CA between the groups. During the 5-year follow-up, the patients with arrhythmia recurrence significantly more often used the OAC, amiodarone, and Ic class of AADs. There was no significant difference in the change in 5-year CHA_2_DS_2_-VASc score (ΔCHA_2_DS_2_-VASc), angiotensin-converting enzyme inhibitor (ACEi), loop diuretics, and mineralocorticoid receptor antagonist (MRA) use after CA between the groups.

### 3.2. Baseline Renal Function

Of 169 patients, 44 (26%) had normal kidney function (eGFR grade 1), 103 (61%) had mildly decreased kidney function (eGFR grade 2), and CKD (eGFR grade 3 or 4) was diagnosed in 22 patients (13%) before ablation. The mean eGFR (CKD-EPI) at baseline was 78.7 ± 17.3 mL/min/1.73 m^2^. There was no significant difference in the mean eGFR and proportion of CKD prior to ablation between the patients with and without post-CA LRAA (Table 1). Prevalence of cardiovascular risk factors in patients with or without CKD before ablation is presented in Table S1 (see Supplementary Materials).

### 3.3. Changes in Renal Function According to the Rhythm Outcome after CA

Linear regression analysis demonstrated that LRAA occurrence after CA was significantly associated with a decrease in post-ablation eGFR, assessed by the CKD-EPI formula (R^2^ 0.07, F 12.639, Beta coefficient −7.636 [95% CI: −11.876 to −3.395], *p* < 0.001). The rhythm outcome after CA was significantly associated with the eGFR change even after adjusting for several clinical variables, including the age, gender, type of AF (PAF vs. NPAF), pre-existing hypertension, and diabetes mellitus (all *p* < 0.001).

### 3.4. Renal Function after 5-Year Follow-Up

In patients with LRAA, the mean eGFR significantly decreased during the 5-year follow-up after CA, regardless of which equation was used for eGFR assessment (for the CKD-EPI eGFR equation: from 78.8 ± 17.3 mL/min/1.73 m^2^ at baseline to 72.0 ± 18.5 mL/min/1.73 m^2^ at final follow-up, *p* < 0.001, and for the MDRD eGFR equation: from 74.8 ± 18.2 mL/min/1.73 m^2^ before the CA to 69.4 ± 19.5 mL/min/1.73 m^2^ after the CA, *p* = 0.006), as presented in Table 2. However, in patients who were LRAA-free after their last CA, the mean eGFR remained stable during the 5 years post procedure (for the CKD-EPI eGFR equation: 78.7 ± 17.3 mL/min/1.73 m^2^ at baseline vs. 79.4 ± 17.4 mL/min/1.73 m^2^ at final follow-up, *p* = NS) or even significantly improved (for the MDRD eGFR equation: from 74.1 ± 17.0 mL/min/1.73 m^2^ before to 77.4 ± 19.6 mL/min/1.73 m^2^ after the procedure, *p* = 0.029).

The 5-year CrCl changes after the CA, calculated by the CG formula, were in line with the previous findings for eGFR, Table 2. There was no change in 5-year renal function in relation to the pre-ablation CT scan (see Supplementary Materials Table S2).

### 3.5. Changes in eGFR Categories

**Figure 2:**
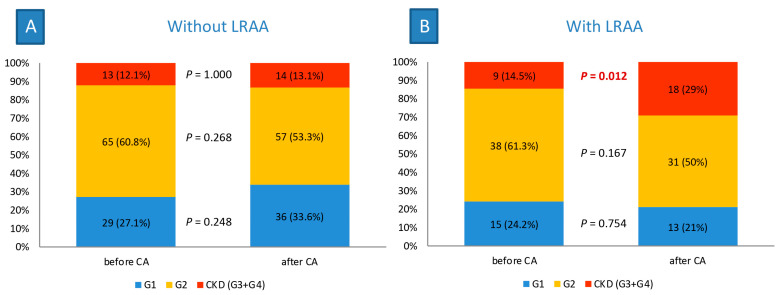
Changes in eGFR categories 5 years after CA: (**A**) in patients without LRAA and (**B**) in patients with LRAA. CA, catheter ablation; CKD, chronic kidney disease; G1, grade 1; G2, grade 2; G3, grade 3; G4, grade 4; LRAA, late recurrence of atrial arrhythmia.

In arrhythmia-free patients after their last CA, there was no significant difference in the eGFR categories before and after CA (Figure 2A). However, patients with LRAA after ablation experienced significant deterioration of their eGFR categories, showing significantly higher prevalence of CKD at the end of follow-up than before CA (29% vs. 14.5%, *p* = 0.012, Figure 2B).

In 105 patients (62.1%), there was no change in eGFR category and they were considered as stable. Among 14 patients (8.3%), with the certain rise in renal function, 13 patients were free from LRAA and one of them experienced LRAA (12.2% vs. 1.6%, *p* = 0.019). Conversely, the certain drop in renal function was observed significantly more frequently in patients with LRAA than in those who were arrhythmia-free (16.1% vs. 3.7%, *p* = 0.008, Table 3). The prevalence of cardiovascular risk factors in patients with or without CKD at 5-year follow-up is presented in Table S3 (see Supplementary Materials).

### 3.6. The Risk Factors for Rapid CKD Progression

The rapid progression of CKD at the 5-year follow-up occurred in 19 patients (11.2%). Univariate analysis showed a significant association between the rapid progression of CKD and female sex, LRAA after last CA and vitamin K antagonist (VKA), amiodarone, loop diuretics, and mineralocorticoid receptor antagonist (MRA) use after the ablation (all *p* < 0.05). Multivariate analysis identified that the female sex (hazard ratio (HR), 3.05; 95% CI, 1.13–8.20; *p* = 0.027), post-ablation LRAA occurrence (HR, 3.36; 95% CI, 1.25–9.06; *p* = 0.016), VKA use post-CA (HR, 3.32; 95% CI, 1.28–8.58; *p* = 0.013), and MRA use after the procedure (HR, 3.28; 95% CI, 1.13–9.54, *p* = 0.029) were independently associated with rapid progression of CKD (Table 4).

## 4. Discussion

The main findings in our study are as follows: (1) LRAA after CA for AF was associated with a significant long-term decrease in renal function, whereas the mean eGFR remained stable or even improved in arrhythmia-free patients; (2) a certain rise in eGFR was observed significantly more frequently in patients who maintained sinus rhythm, while a certain drop in eGFR occurred significantly more often in those patients with LRAA post-CA; (3) LRAA after last CA, female sex, and VKA and MRA use during the follow-up after CA were independent risk factors for rapid progression of CKD.

There are several pathophysiological mechanisms connecting AF and CKD, such as activation of the renin-angiotensin-aldosterone system (RAAS), inflammation, and oxidative stress [4,12,13,14,15]. The presence of AF results in the loss of LA systole, reduction in cardiac output, and increases the risk of renal micro-thrombosis. These neurohumoral and hemodynamic effects cause a reduction in kidney perfusion pressure, leading to a decrease in eGFR [16]. Therefore, successful rhythm control after AF ablation may improve renal function [15,17].

Previous studies identified AF recurrence after CA as a predictor of eGFR reduction [15,17,18]. Most of these studies evaluated renal function change within 1 to 2 years after CA [17,19,20]. In the long-term 5-year follow-up study, the CA of AF, but not pharmacological therapy, significantly improved eGFR, and this improvement was more pronounced in the patients who remained in sinus rhythm after the CA and in non-diabetic patients [15]. In a more recent long-term post-CA AF study, the decline in eGFR > 30% was significantly associated with AF recurrence, diabetes mellitus, congestive heart failure, and pre-ablation history of CKD [18].

Estimated GFR declines with aging by 0.3–1 mL/min/1.73 m^2^ per year in the general population and by 1–2.65 mL/min/1.73 m^2^ per year in patients with established CKD [21,22,23,24,25]. To reduce the impact of small and expected changes in eGFR during the 5-year follow-up, we used KDIGO definitions of a certain rise and certain drop in eGFR and rapid progression of CKD. The CG formula for creatinine clearance is recommended to determine a renal indication for dose reduction of the direct oral anticoagulants (DOACs), while MDRD and CKD-EPI are the two most commonly used formulas to estimate GFR in clinical practice [26]. In our study, we demonstrated the concordance between these three formulas in patients who underwent CA of AF. The only exception was that using MDRD, but not CKD-EPI or CG, showed significantly improved renal function 5 years after the procedure in patients who maintained sinus rhythm.

In the present study, apart from LRAA after last CA, other independent risk factors for rapid progression of CKD were female sex and VKA and MRA use after CA of AF. Previous studies and meta-analyses provided the opposite conclusions regarding the effect of gender on kidney disease progression [27]. In most population-based studies, the renal function decline was faster in men than in women [22,28]. However, a meta-analysis of 11 randomized trials, mostly including postmenopausal women, showed that renal disease progression might even be faster in women than in men [29]. In our study, female patients at ablation were significantly older than male patients, had a higher prevalence of hypertension at baseline, and more often experienced AF recurrence after CA procedure (44.6% vs. 31.7%, see Supplementary Materials Table S4).

Activation of the RAAS is a major factor in pathogenesis and progression of CKD by inducing inflammation and fibrosis [4]. The use of MRA reduces proteinuria, but increases the risk of hyperkalemia [30]. In our study, patients on MRA therapy had a significantly higher prevalence of comorbidities that could promote the progression of CKD, such as NPAF, hypertension, congestive heart failure, coronary artery disease, and concomitant use of loop diuretics during follow-up (see Supplementary Materials Table S5).

As expected, in the present study, we found that patients with LRAA after the CA procedure more frequently used VKA, DOAC, and AADs during follow-up compared with arrhythmia-free patients. However, only VKA use was independently associated with the rapid progression of CKD. Previous studies demonstrated that patients on warfarin had an increased vascular calcification mediated through inactivation of vitamin-K-dependent matrix Gla protein [31,32,33]. In a large cohort of warfarin users, 33% of patients with CKD developed warfarin-related nephropathy (WRN), which is characterized by acute kidney injury (AKI) owing to tubular obstruction by red blood cell casts in patients with an acute increase in the international normalized ratio (INR) > 3 [34]. Although it was more frequent in patients with CKD, WRN also occurred in 16.5% patients without CKD and was associated with increased mortality [34,35,36,37]. In the present study, there was no significant difference in clinical characteristics between patients using VKA and DOAC (see Supplementary Materials Table S6).

### Study Limitations

The atrial fibrillation recurrence rate was probably underestimated using intermittent rhythm monitoring by 24 h Holter recordings in our study, but this strategy is in line with a usual clinical practice and the current guidelines [1].

We evaluated renal function using a single sCr value 5 years after the initial CA procedure. Annual measurement of eGFR and albuminuria during follow-up would provide a better assessment of kidney function and CKD progression.

The majority of patients in our study had a normal renal function (26%) or had only a slightly reduced renal function (61%), while the proportion of patients with moderate or severe CKD was low (13%). Therefore, it has not been clarified whether ablation could have a beneficial effect on improving renal function in patients with the late stage of renal failure. However, our data might suggest that an early intervention by CA could protect renal function in AF patients with normal eGFR or in those with earlier stages of kidney disease.

## 5. Conclusions

In our study, LRAA after last CA was associated with a significant decrease in eGFR, as well as a certain drop in kidney function, and was an independent risk factor for rapid CKD progression. Conversely, eGFR in arrhythmia-free patients remained stable or even improved significantly after CA procedure. The patients after successful CA of AF significantly more often demonstrated a certain rise in kidney function.

## Figures and Tables

**Table 1:** Clinical characteristics of the study population.

	All Patients, *n* = 169	No LRAA after Last CA, *n* = 107 (63.3%)	LRAA after Last CA, *n* = 62 (36.7%)	*p*-Value
Age, y	59.6 ± 10.1	58.8 ± 10.7	61.0 ± 8.8	0.181
Male patients	104 (61.5%)	71 (66.4%)	33 (53.2%)	0.091
BMI, kg/m^2^	27.1 ± 4.0	26.7 ± 4.1	27.7 ± 3.7	0.107
NPAF	39 (23.1%)	16 (15%)	23 (37.1%)	0.001 *
History of AF before CA, y	4.0 (2.0–7.0)	4.0 (2.0–7.0)	4.0 (2.0–7.0)	0.971
Failed AADs, *n*	1.6 ± 0.7	1.5 ± 0.7	1.7 ± 0.7	0.139
Hypertension	117 (69.2%)	72 (67.3%)	45 (72.6%)	0.473
Diabetes mellitus	17 (10.1%)	9 (8.4%)	8 (12.9%)	0.349
Congestive heart failure	10 (5.9%)	5 (4.7%)	5 (8.1%)	0.368
Stroke	12 (7.1%)	6 (5.6%)	6 (9.7%)	0.321
Coronary artery disease	13 (7.7%)	6 (5.6%)	7 (11.3%)	0.181
BUN (mmol/L)	6.1 ± 1.9	6.2 ± 1.8	6.0 ± 2.1	0.431
Serum creatinine (µmol/L)	86.1 ± 20.6	87.8 ± 20.5	83.3 ± 20.5	0.175
Baseline eGFR (CKD-EPI), mL/min per 1.73 m^2^	78.7 ± 17.3	78.7 ± 17.3	78.8 ± 17.3	0.973
GFR category 1	44 (26%)	29 (27.1%)	15 (24.2%)	0.678
GFR category 2	103 (61%)	65 (60.8%)	38 (61.3%)	0.944
CKD (GFR category ≥ 3)	22 (13%)	13 (12.1%)	9 (14.5%)	0.659
CHA2DS2-VASc score	1.8 ± 1.3	1.6 ± 1.1	2.2 ± 1.5	0.016 *
ΔCHA2DS2-VASc score	0.4 ± 0.7	0.4 ± 0.7	0.4 ± 0.6	0.563
LA diameter, mm	41.6 ± 4.8	40.7 ± 4.8	43.0 ± 4.6	0.002 *
LV EDD, mm	52.7 ± 4.3	52.8 ± 4.1	52.7 ± 4.6	0.900
LV EF, %	59.7 ± 7.3	60.3 ± 6.6	58.6 ± 8.3	0.154
LV EF < 50%	13 (7.7%)	6 (5.6%)	7 (11.3%)	0.181
Cardiac CT scan before ablation	149 (88.2%)	92 (86%)	57 (91.9%)	0.248
VKA	44 (26%)	20 (18.7%)	24 (38.7%)	0.004 *
DOACs	62 (36.7%)	30 (28%)	32 (51.6%)	0.002 *
Amiodarone	44 (26%)	15 (14%)	29 (46.8%)	<0.001 *
Propafenone	26 (15.4%)	12 (11.2%)	14 (22.6%)	0.048 *
Flecainide	36 (21.3%)	16 (15%)	20 (32.3%)	0.008 *
Beta blockers	137 (81.1%)	84 (78.5%)	53 (85.5%)	0.264
ACEi	84 (49.7%)	49 (45.8%)	35 (56.5%)	0.182
ARB	19 (11.2%)	12 (11.2%)	7 (11.3%)	0.988
Thiazide diuretics	36 (21.3%)	23 (21.5%)	13 (21%)	0.936
Loop diuretics	21 (12.4%)	12 (11.2%)	9 (14.5%)	0.531
MRA	16 (9.5%)	9 (8.4%)	7 (11.3%)	0.538
statins	50 (29.6%)	27 (25.2%)	23 (37.1%)	0.103

Data are presented as mean ± 1 SD, median (IQR), or as numbers (percentages). AADs, antiarrhythmic drugs; ACEi, angiotensin-converting enzyme inhibitors; AF, atrial fibrillation; ARB, angiotensin receptor blockers; BMI, body mass index; BUN, blood urea nitrogen; CA, catheter ablation; CKD, chronic kidney disease; CKD-EPI, chronic kidney disease epidemiology collaboration; CT, computed tomography; DOACs, direct oral anticoagulants; EDD, end-diastolic diameter; EF, ejection fraction; eGFR, estimated glomerular filtration rate; LA, left atrium; LRAA, late recurrence of atrial arrhythmia; LV, left ventricle; MRA, mineralocorticoid receptor antagonists; NPAF, nonparoxysmal atrial fibrillation; VKA, vitamin K antagonists. * *p* < 0.05.

**Table 2:** Changes in eGFR and creatinine clearance 5 years after CA according to the rhythm outcome.

Renal Function	LRAA after Last CA	Before CA	Post CA	*p*-Value (Post CA vs. before CA)	Δ (Post CA– before CA), *n* (95% CI)	*p*-Value (LRAA vs. No LRAA)
CKD-EPI eGFR, mL/min per 1.73 m^2^	LRAA	78.8 ± 17.3	72.0 ± 18.5	<0.001 *	−6.8 (−10.2 to −3.4)	<0.001 *
	No LRAA	78.7 ± 17.3	79.4 ± 17.4	0.555	0.7 (−1.8 to 3.3)	
MDRD eGFR, mL/min per 1.73 m^2^	LRAA	74.8 ± 18.2	69.4 ± 19.5	0.006 *	−5.4 (−9.2 to 1.6)	<0.001 *
	No LRAA	74.1 ± 17.0	77.4 ± 19.6	0.029 *	3.3 (0.4 to 6.3)	
Creatinine clearence, mL/min	LRAA	98.2 ± 31.2	87.6 ± 31.3	<0.001 *	−10.6 (−15.8 to −5.3)	<0.001 *
	No LRAA	94.0 ± 24.7	94.8 ± 26.2	0.624	0.8 (−2.5 to 4.1)	
Serum creatinine (µmol/L)	LRAA	83.3 ± 20.5	89.2 ± 26.1	0.006 *	5.9 (1.7 to 10.2)	<0.001 *
	No LRAA	87.8 ± 20.5	83.8 ± 19.0	0.023 *	−4.0 (−7.4 to −0.6)	
BUN (mmol/L)	LRAA	6.0 ± 2.1	6.5 ± 2.7	0.078	0.5 (−0.1 to 1.1)	0.006 *
	No LRAA	6.2 ± 1.8	5.8 ± 1.6	0.038 *	−0.3 (−0.7 to −0.1)	

Data are presented as mean ± 1 SD. BUN, blood urea nitrogen; CA, catheter ablation; CKD-EPI, Chronic Kidney Disease Epidemiology Collaboration; eGFR, estimated glomerular filtration rate; LRAA, late recurrence of atrial arrhythmia; MDRD, modification of diet in renal disease. * *p* < 0.05.

**Table 3:** Progression of CKD during follow-up in relation to the rhythm outcome after CA.

	All Patients, *n* = 169	No LRAA after Last CA, *n* = 107 (63.3%)	LRAA after Last CA, *n* = 62 (36.7%)	*p*-Value
Certain rise	14 (8.3%)	13 (12.2%)	1 (1.6%)	0.019 *
Uncertain rise	17 (10.1%)	12 (11.2%)	5 (8.1%)	0.512
Stable	105 (62.1%)	65 (60.7%)	40 (64.5%)	0.626
Uncertain drop	19 (11.2%)	13 (12.2%)	6 (9.7%)	0.624
Certain drop	14 (8.3%)	4 (3.7%)	10 (16.1%)	0.008 *

Data are presented as numbers (percentages). CA, catheter ablation; LRAA, late recurrence of atrial arrhythmia. * *p* < 0.05.

**Table 4:** Risk factors for rapid progression of CKD.

	Univariate Analysis		Multivariate Analysis	
	HR (95% CI)	*p*-Value	HR (95% CI)	*p*-Value
Age	1.04 (0.98–1.09)	0.197		
Female patients	3.31 (1.30–8.44)	0.012 *	3.05 (1.13–8.20)	0.027 *
BMI	1.02 (0.92–1.13)	0.743		
NPAF	1.17 (0.42–3.27)	0.763		
Hypertension	1.46 (0.52–4.05)	0.451		
Diabetes mellitus	1.74 (0.50–5.98)	0.378		
Coronary artery disease	2.18 (0.49–9.62)	0.303		
CHA2DS2-VASc score	1.16 (0.84–1.59)	0.360		
ΔCHA2DS2-VASc score	1.38 (0.75–2.56)	0.299		
baseline eGFR (CKD-EPI), mL/min per 1.73 m^2^	1.02 (0.99–1.04)	0.219		
LA diameter, mm	1.02 (0.93–1.12)	0.638		
LV EDD, mm	0.95 (0.85–1.06)	0.376		
LV EF, %	1.01 (0.94–1.08)	0.889		
LV EF < 50%	0.90 (0.12–6.81)	0.917		
VKA	3.52 (1.41–8.75)	0.007 *	3.32 (1.28–8.58)	0.013 *
DOACs	1.15 (0.45–2.93)	0.766		
Amiodarone	2.59 (1.05–6.39)	0.038 *	1.14 (0.44–2.99)	0.785
Propafenone	1.86 (0.67–5.16)	0.237		
Flecainide	1.51 (0.54–4.20)	0.432		
Beta blockers	1.10 (0.36–3.34)	0.862		
ACEi	1.08 (0.44–2.66)	0.870		
ARB	2.67 (0.96–7.43)	0.060		
ACEi/ARB	2.09 (0.75–5.84)	0.157		
Thiazide diuretics	0.90 (0.29–2.72)	0.846		
Loop diuretics	3.22 (1.22–8.50)	0.018 *	1.55 (0.41–5.83)	0.516
MRA	3.99 (1.43–11.15)	0.008 *	3.28 (1.13–9.54)	0.029 *
statins	1.04 (0.40–2.75)	0.934		
LRAA after last CA	4.54 (1.72–11.98)	0.002 *	3.36 (1.25–9.06)	0.016 *

ACEi, angiotensin-converting enzyme inhibitors; ARB, angiotensin receptor blockers; BMI, body mass index; CA, catheter ablation; CI, confidence interval; CKD-EPI, chronic kidney disease epidemiology collaboration; DOACs, direct oral anticoagulants; EDD, end-diastolic diameter; EF, ejection fraction; eGFR, estimated glomerular filtration rate; HR, hazard ratio; LA, left atrium; LRAA, late recurrence of atrial arrhythmia; LV, left ventricle; MRA, mineralocorticoid receptor antagonists; NPAF, nonparoxysmal atrial fibrillation; VKA, vitamin K antagonists. * *p* < 0.05.

## Data Availability

The data that support the findings of this study are available from the corresponding author upon reasonable request.

## Supplementary Materials

The following supporting information can be downloaded at https://www.mdpi.com/article/10.3390/jcdd10040151/s1. Table S1: Prevalence of cardiovascular risk factors in patients with or without CKD at baseline; Table S2: Changes in 5-year renal function in relation to the pre-ablation CT scan; Table S3: Prevalence of cardiovascular risk factors in patients with or without CKD at 5-year follow-up; Table S4: Differences in clinical characteristics according to gender; Table S5: Differences in clinical characteristics based on the use of MRA during follow-up; Table S6: Differences in clinical characteristics in relation to the type of OAC during follow-up.
