# Supplementary material for: Long-Term Renal Function after Catheter Ablation of Atrial Fibrillation
Source: J Cardiovasc Dev Dis. 2023 Mar 31;10(4):151. doi: 10.3390/jcdd10040151 (PMC10142031; doi:10.3390/jcdd10040151)
Supplement: Supplementary file 1 [file jcdd-10-00151-s001.zip › jcdd-2284076-supplementary.pdf]

Supplementary materials

Table S1. Prevalence of cardiovascular risk factors in patients with or without CKD at baseline

	All patients, n=169	Patients with CKD, n=22 (13%)	Patients without CKD, n=147 (87%)	P value
Hypertension	117 (69.2%)	18 (81.8%)	99 (67.3%)	0.170
Diabetes mellitus	17 (10.1%)	5 (22.7%)	12 (8.2%)	0.034*
Congestive heart failure	10 (5.9%)	3 (13.6%)	7 (4.8%)	0.100

Data are presented as numbers (percentages). CKD indicates chronic kidney disease.

Table S2. Changes in 5-year renal function in relation to the pre-ablation CT scan.

Renal function	Cardiac CT scan before CA	Before CA	Post CA	P value (Post CA vs Before CA)	Δ (Post CA-Before CA), n (95% CI)	P value (CT scan vs No CT scan)
CKD-EPI eGFR, ml/min per 1.73 m2	CT scan	79.3±16.5	77.3±17.9	0.090	-2.0 (-4.3 to 0.3)	0.942
	No CT scan	74.0±22.0	71.8±19.1	0.423	-2.2 (-7.8 to 3.4)	

Data are presented as mean ± 1 SD. CA indicates catheter ablation, CKD, chronic kidney disease; CKD-EPI, chronic kidney disease epidemiology collaboration; CT, computed tomography; eGFR, estimated glomerular filtration rate;

Table S3. Prevalence of cardiovascular risk factors in patients with or without CKD at 5-year follow-up

	All patients, n=169	Patients with CKD, n=32 (18.9%)	Patients without CKD, n=137 (81.1%)	P value
Hypertension	125 (74%)	27 (84.4%)	98 (71.5%)	0.136
Diabetes mellitus	23 (13.6%)	6 (18.8%)	17 (12.4%)	0.346
Congestive heart failure	14 (8.3%)	8 (25%)	6 (4.4%)	<0.001*

Data are presented as numbers (percentages). CKD indicates chronic kidney disease.

Table S4. Differences in clinical characteristics according to gender

	All patients, n=169	Male, n=104 (61.5%)	Female, n=65 (38.5%)	*P* value
Age, y	59.6±10.1	57.9±9.5	62.3±10.4	0.005*
BMI, kg/m2	27.1±4.0	27.1±3.2	27.0±5.1	0.886
NPAF	39 (23.1%)	24 (23.1%)	15 (23.1%)	1.000
History of AF before CA, y	5.4±4.5	5.6±4.9	4.9±3.8	0.318
Failed AADs, n	1.6±0.7	1.6±0.7	1.6±0.7	0.959
Hypertension	117 (69.2%)	66 (63.5%)	51 (78.5%)	0.040*
Diabetes mellitus	17 (10.1%)	9 (8.7%)	8 (12.3%)	0.442
Congestive heart failure	10 (5.9%)	8 (7.7%)	2 (3.1%)	0.320
Stroke	12 (7.1%)	7 (6.7%)	5 (7.7%)	0.813
Coronary artery disease	13 (7.7%)	10 (9.6%)	3 (4.6%)	0.374
CHA2DS2-VASc score	1.8±1.3	1.3±1.2	2.6±1.1	<0.001*
baseline eGFR (CKD-EPI), ml/min per 1.73 m2	78.7±17.3	80.3±17.5	76.2±16.7	0.133
CKD (GFR category≥ 3)	22 (13%)	11 (10.6%)	11 (16.9%)	0.233
LA diameter, mm	41.6±4.8	42.6±4.3	39.9±5.2	<0.001*
LV EDD, mm	52.7±4.3	54.1±4.1	50.6±3.8	<0.001*
LV EF, %	59.7±7.3	59.4±7.6	60.0±6.8	0.569
LV EF <50%	13 (7.7%)	9 (8.7%)	4 (6.2%)	0.768
LRAA after last CA	62 (36.7%)	33 (31.7%)	29 (44.6%)	0.091

Data are presented as mean ± 1 SD or as numbers (percentages). AADs indicates antiarrhythmic drugs; AF, atrial fibrillation; BMI, body mass index; CA, catheter ablation; CKD, chronic kidney disease; CKD-EPI, chronic kidney disease epidemiology collaboration; EDD, end-diastolic diameter; EF, ejection fraction; eGFR, estimated glomerular filtration rate; LA, left atrium; LRAA, late recurrence of atrial arrhythmia; LV, left ventricle; NPAF, nonparoxysmal atrial fibrillation; *$P<0.05$

Table S5. Differences in clinical characteristics based on the use of MRA during follow-up

	All patients, n=169	MRA during follow-up, n=16 (9.5%)	Without MRA during follow-up, n=153 (90.5%)	P value
Age, y	59.6±10.1	60.5±11.8	59.5±9.9	0.709
Male patients	104 (61.5%)	7 (43.8%)	97 (63.4%)	0.124
BMI, kg/m2	27.1±4.0	28.6±5.1	26.9±3.8	0.102
NPAF	39 (23.1%)	8 (50%)	31 (20.3%)	0.007*
History of AF before CA, y	5.4±4.5	5.2±4.5	5.4±4.5	0.863
Failed AADs, n	1.6±0.7	1.4±0.5	1.6±0.7	0.176
Hypertension	117 (69.2%)	15 (93.8%)	102 (66.7%)	0.024*
Diabetes mellitus	17 (10.1%)	3 (18.8%)	14 (9.2%)	0.206
Congestive heart failure	10 (5.9%)	4 (25%)	6 (3.9%)	0.008*
Stroke	12 (7.1%)	1 (6.3%)	11 (7.2%)	1.000
Coronary artery disease	13 (7.7%)	4 (25%)	9 (5.9%)	0.023*
CHA2DS2-VASc score	1.8±1.3	2.8±1.2	1.7±1.3	0.002*
baseline eGFR (CKD-EPI), ml/min per 1.73 m2	78.7±17.3	70.9±19.6	79.5±16.9	0.057
CKD (GFR category≥ 3)	22 (13%)	4 (25%)	18 (11.8%)	0.231
LV EF, %	59.7±7.3	52.4±11.0	60.4±6.4	0.011*
LV EF <50%	13 (7.7%)	6 (37.5%)	7 (4.6%)	<0.001*
LRAA after last CA	62 (36.7%)	7 (43.8%)	55 (35.9%)	0.538
Loop diuretics	21 (12.4%)	13 (81.3%)	8 (5.2%)	<0.001*

Data are presented as mean ± 1 SD or as numbers (percentages). AADs indicates antiarrhythmic drugs; AF, atrial fibrillation; BMI, body mass index; CA, catheter ablation; CKD, chronic kidney disease; CKD-EPI, chronic kidney disease epidemiology collaboration; EF, ejection fraction; eGFR, estimated glomerular filtration rate; LRAA, late recurrence of atrial arrhythmia; LV, left ventricle; NPAF, nonparoxysmal atrial fibrillation; *P<0.05

Table S6. Differences in clinical characteristics in relation to the type of OAC during follow-up

	VKA during follow-up, n=44	DOAC during follow-up, n=62	P value
Age, y	61.5±8.2	62.8±8.8	0.446
Male patients	26 (59.1%)	32 (51.6%)	0.446
BMI, kg/m2	27.1±4.0	28.0±4.1	0.247
NPAF	16 (36.4%)	16 (25.8%)	0.243
History of AF before CA, y	6.5±5.4	4.7±4.0	0.065
Failed AADs, n	1.7±0.6	1.6±0.7	0.573
Hypertension	33 (75%)	44 (71%)	0.646
Diabetes mellitus	5 (11.4%)	8 (12.9%)	0.812
Congestive heart failure	3 (6.8%)	3 (4.8%)	0.691
Stroke	5 (11.4%)	5 (8.1%)	0.567
Coronary artery disease	4 (9.1%)	6 (9.7%)	1.000
CHA2DS2-VASc score	2.0±1.3	2.2±1.4	0.469
baseline eGFR (CKD-EPI), ml/min per 1.73 m2	77.6±16.6	73.2±16.2	0.182
CKD (GFR category≥ 3)	5 (11.4%)	11 (17.7%)	0.366
LA diameter, mm	43.0±4.5	42.3±4.8	0.452
LV EDD, mm	52.8±5.1	52.8±4.1	0.964
LV EF, %	57.8±7.1	60.2±7.2	0.092
LV EF <50%	3 (6.8%)	6 (9.7%)	0.732
LRAA after last CA	24 (54.5%)	32 (51.6%)	0.766
Amiodarone	19 (43.2%)	21 (33.9%)	0.330
Propafenon	10 (22.7%)	14 (22.6%)	0.986
Flecainide	7 (15.9%)	21 (33.9%)	0.039*
ACEi/ARB	29 (65.9%)	43 (69.4%)	0.708
Thiazide diuretics	5 (11.8%)	19 (30.6%)	0.019*
Loop diuretics	8 (18.2%)	10 (16.1%)	0.782
MRA	7 (15.9%)	6 (9.7%)	0.335
statins	16 (36.4%)	21 (33.9%)	0.791

Data are presented as mean ± 1 SD or as numbers (percentages). AADs indicates antiarrhythmic drugs; ACEi, angiotensin-converting enzyme inhibitors; AF, atrial fibrillation; ARB, angiotensin receptorblockers; BMI, body mass index; CA, catheter ablation; CKD, chronic kidney disease; CKD-EPI, chronic kidney disease epidemiology collaboration; DOAC, direct oral anticoagulants; EDD, end-diastolic diameter; EF, ejection fraction; eGFR, estimated glomerular filtration rate; LA, left atrium; LRAA, late recurrence of atrial arrhythmia; LV, left ventricle; MRA, mineralocorticoid receptor antagonists; NPAF, nonparoxysmal atrial fibrillation; OAC, oral anticoagulants; VKA, vitamin K antagonists; *P<0.05
